# Rhythmic EEG patterns in extremely preterm infants: Classification and association with brain injury and outcome

**DOI:** 10.1016/j.clinph.2017.08.035

**Published:** 2017-12

**Authors:** Lauren C. Weeke, Inge M. van Ooijen, Floris Groenendaal, Alexander C. van Huffelen, Ingrid C. van Haastert, Carolien van Stam, Manon J. Benders, Mona C. Toet, Lena Hellström-Westas, Linda S. de Vries

**Affiliations:** a Department of Neonatology, Wilhelmina Children’s Hospital, University Medical Center Utrecht, The Netherlands; b Brain Center Rudolf Magnus, University Medical Center Utrecht, The Netherlands; c Department of Clinical Neurophysiology, University Medical Center Utrecht, The Netherlands; d Department of Clinical Psychology, University Medical Center Utrecht, The Netherlands; e Department of Women’s and Children’s Health, Uppsala University, Uppsala, Sweden

**Keywords:** EEG, Seizures, Periodic epileptiform discharges, PED, Preterm infant, Brain injury, Outcome

## Abstract

**Objective:**

Classify rhythmic EEG patterns in extremely preterm infants and relate these to brain injury and outcome.

**Methods:**

Retrospective analysis of 77 infants born <28 weeks gestational age (GA) who had a 2-channel EEG during the first 72 h after birth. Patterns detected by the BrainZ seizure detection algorithm were categorized: ictal discharges, periodic epileptiform discharges (PEDs) and other waveforms. Brain injury was assessed with sequential cranial ultrasound (cUS) and MRI at term-equivalent age. Neurodevelopmental outcome was assessed with the BSITD-III (2 years) and WPPSI-III-NL (5 years).

**Results:**

Rhythmic patterns were observed in 62.3% (ictal 1.3%, PEDs 44%, other waveforms 86.3%) with multiple patterns in 36.4%. Ictal discharges were only observed in one and excluded from further analyses. The EEG location of the other waveforms (*p* < 0.05), but not PEDs (*p* = 0.238), was significantly associated with head position. No relation was found between the median total duration of each pattern and injury on cUS and MRI or cognition at 2 and 5 years.

**Conclusions:**

Clear ictal discharges are rare in extremely preterm infants. PEDs are common but their significance is unclear. Rhythmic waveforms related to head position are likely artefacts.

**Significance:**

Rhythmic EEG patterns may have a different significance in extremely preterm infants.

## Highlights

• Most rhythmic EEG patterns in extremely preterm infants related to head position.
• Clear ictal discharges were only observed in one out of 77 infants (1.3%).
• PEDs were prevalent, but their significance is not known.
• PEDs were not related to brain injury or poor cognition.

## 1 Introduction

In many units, neuro-monitoring with electroencephalography (EEG) during the first postnatal days has become part of standard care. Brain protection has become one of the main aims of neonatal intensive care, since the survival rate of extremely preterm infants (born <28 weeks gestational age) has increased due to major advances in perinatal care (Costeloe et al., 2012, Zegers et al., 2016).

Seizures have been related to adverse outcome in preterm infants (Davis et al., 2011, Pisani et al., 2008, Pisani et al., 2004, Shah et al., 2010, Vesoulis et al., 2014). The incidence of seizures in preterm infants has been estimated at 4–48% (Scher et al., 1993, Vesoulis et al., 2014). This is higher than reported in full-term infants (Scher et al., 1993), while the response rate to antiepileptic drugs (AEDs) seems to be significantly lower in preterm infants (Weeke et al., 2016). The majority of seizures are subclinical, requiring continuous EEG for seizure detection (Patrizi et al., 2003). In the neonatal intensive care unit (NICU), detection of subclinical seizure patterns can be aided by automatic seizure detection algorithms (Navakatikyan et al., 2006).

However, little is known about EEG seizure morphology in extremely preterm infants. Clinicians are faced with a wide range of rhythmic EEG patterns that may be recognized by seizure detection algorithms as subclinical seizures. However, not all patterns seem to comply with the definition of an electroencephalographic seizure, which is rhythmic activity, lasting for at least 10 s, with a clear beginning, middle and end, and showing evolution in amplitude, frequency and/or morphology (Husain, 2005). This raises the question whether these patterns are subclinical seizures that require treatment and are related to poor outcome or whether they accompany normal brain development.

The aim of the present study was to classify all rhythmic EEG patterns, recognized by the BrainZ seizure detection algorithm, based on morphology and relate these different patterns to brain injury and neurodevelopmental outcome in a cohort of extremely preterm infants who were monitored with 2-channel EEG during the first 72 h after birth as standard of care. Our hypothesis was that ictal discharges and periodic epileptiform discharges (PEDs) would be associated with brain injury and poor cognitive outcome.

## 2 Methods

### 2.1 Patients

All infants born <28 weeks gestational age (GA) between May 2008 and December 2010 who had been admitted to the level III NICU of the Wilhelmina Children’s Hospital in Utrecht, the Netherlands, and had a 2-channel EEG recording during the first 72 h after birth were retrospectively analyzed. Sixty-three infants were participants of a European multicenter study (Neonatal Estimation of Brain Damage Risk and Identification of Neuroprotectants [NeoBrain]) (Dammann et al., 2007). Clinical data, such as GA, birth weight, Apgar score, head position (side the infant’s head was turned to) and morphine and AED administration, was obtained from the electronically available medical records. Head position was recorded in the electronic medical records by the nurses every time the infant’s position was changed. Permission from the medical ethics committee and parental informed consent were obtained.

### 2.2 EEG monitoring

Patients were monitored using the BrainZ Monitor (BRM2 or 3 version, Natus, Seattle, USA). It records a two-channel amplitude-integrated EEG as well as a raw EEG from two needle electrodes over each hemisphere (F4-P4, F3-P3, according to the international 10–20 system of electrode placement modified for neonates) and has a built-in seizure detection algorithm (Navakatikyan et al., 2006).

### 2.3 EEG patterns

At the markers placed by the seizure detection algorithm of the BrainZ Monitor, the raw EEG was visually analyzed and categorized by consensus reading of five observers (AH, IO, LV, LH, LW). Rhythmic patterns were categorized into five different categories: ictal discharges, PEDs, PED-like waves, zeta waves and sinusoidal waves. These categories were based on patterns described in older children and adults (Deuschl and Eisen, 1999, Noachtar et al., 1999). The patterns that did not fit into these five categories or were clear artefacts caused by high-frequency ventilation or electrocardiography were discarded. For each EEG pattern that could be categorized into one of the five categories, the total duration in seconds (burden), the mean wavelength in seconds and the location on the EEG (left, right or bilateral) was determined for each infant.

**Fig. 1:**
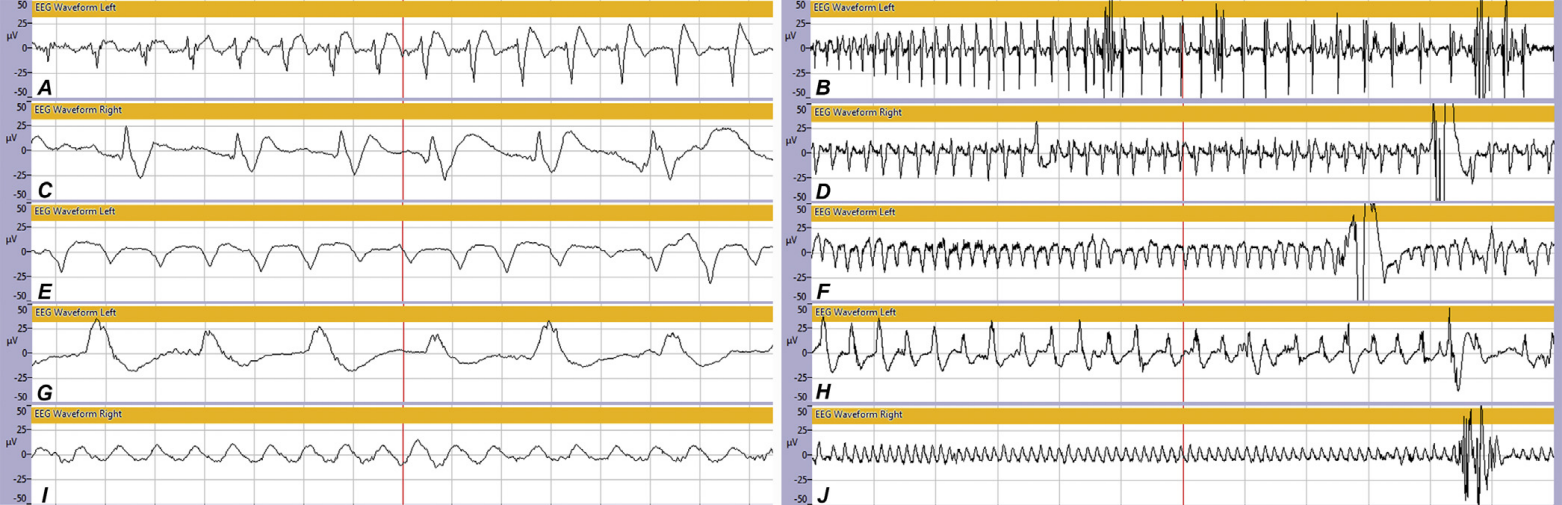
Examples of the different EEG patterns at 15 s (A, C, E, G, I) and 1 min per screen (B, D, F, H, J) that were detected in this study by the seizure detection algorithm of the BrainZ Monitor as indicated by the orange markers. Ictal discharges (A, B), periodic epileptiform discharges (PEDs) (C, D), PED-like waves (E, F), zeta waves (G, H) and sinusoidal waves (I, J).

*Ictal discharges:* have been described in neonates as rhythmic activity, lasting for at least 10 s, with a clear beginning, middle and end, and showing evolution in amplitude, frequency and/or morphology (Fig. 1A and B) (Husain, 2005). This activity can consist of spikes, sharp waves, spike-wave complexes or rhythmic delta or theta waves (Andre et al., 2010).

*Periodic epileptiform discharges (PEDs):* have been described in older infants and adults as sharp waves followed by a pronounced incision and a slow wave. These complexes repeat every 0.5–4 s, but do not show evolution (Fig. 1C and D) (Brenner and Schaul, 1990). They have been associated with brain lesions, epilepsy and poor outcome in children and adults (Chong and Hirsch, 2005, Hamano et al., 1994, Yemisci et al., 2003).

*PED-like waves:* these complexes were detected in our cohort but could not be related to a category described in the literature. They might be considered as PEDs in which the sharp waves are lacking. They consist of a positive sharp deflection from baseline forming an incision, followed by a periodic slow wave of generally more than one second (Fig. 1E and F).

*Zeta waves:* as described by Magnus and van der Holst (1987) consist of a slow wave with a rapid negative first phase, followed by a relatively slower positive second phase crossing the baseline. This wave is followed by a slow negative wave returning to baseline. Thus a Z-shaped complex appears with a duration of more than one second. Such complexes generally occur in trains of several seconds (Fig. 1G and H). In adults, zeta waves were associated with brain lesions with acute onset, such as intracranial hemorrhages (Magnus and van der Holst, 1987).

*Sinusoidal waves:* these waves resemble sine waves. They may occur in different, but generally low delta frequencies (Fig. 1I and J). In older children and adults frontal and occipital intermittent rhythmic delta waves have been described and related to deeply localized brain abnormalities (Watemberg et al., 2007, Watemberg et al., 2002) Sine waves were also thought to initiate seizures and metamorphose into other patterns as described by Blume et al. (1984).

### 2.4 Neuro-imaging

Cranial ultrasonography (cUS) was performed on admission to the NICU and repeated every day for the first 72 h and at weekly intervals thereafter until discharge. Magnetic resonance imaging (MRI) on a 3 T MR system was performed at term-equivalent age (39–43 weeks). The scanning protocol included T1-, T2-, diffusion- and susceptibility-weighted images. For each infant, it was determined whether abnormalities were seen on cUS or MRI and how severe these abnormalities were. Injury severity was categorized as mild (cUS: intraventricular hemorrhage [IVH] grade 1–2 according to Papile et al. (1978); MRI: IVH grade 1–2, <6 punctate white matter lesions [PWML], <6 punctate cerebellar lesions), moderate (cUS: IVH grade 3; MRI: IVH grade 3, ≥6 PWML, ≥6 punctate cerebellar lesions or a large unilateral lesion [<50% of the hemisphere]) or severe (cUS: IVH grade 4, infarction, periventricular hemorrhagic infarction, perforator stroke, cystic periventricular leukomalacia [cPVL], (de Vries et al., 1992) post-hemorrhagic ventricular dilatation [PHVD]; MRI: periventricular hemorrhagic infarction, cPVL (Martinez-Biarge et al., 2016), PHVD, large cerebellar hemorrhage [>50% of hemisphere]). The Kidokoro global score was calculated based on the MRI at term-equivalent age as a marker for injury severity and maturation (Kidokori et al., 2014).

### 2.5 Neurodevelopmental outcome

The Bayley Scales of Infant and Toddler Development, third edition (BSITD-III) was used to assess cognitive outcome at 30 months corrected age (corrected for GA at birth), by calculating the cognitive composite score (Bayley, 2006). The Wechsler Preschool and Primary Scale of Intelligence, third edition, Dutch version (WPPSI-III-NL) was used to assess the intelligence quotient (IQ) at 5 years of age (Wechsler, 2010). The mean (standard deviation [SD]) in a normative population is 100 (15) for the cognitive composite score and the IQ.

### 2.6 Statistical analysis

SPSS version 21 (IBM Corp., Armonk, NY, USA) was used to perform statistical analyses. Chi-squared or Fisher’s exact test was used to investigate the relation between the location of the EEG patterns (right or left) and the infant’s head position (right or left) and compare the proportion of infants with each pattern between those with and without injury on cUS during the first 72 h after birth or on MRI at term-equivalent age and between deceased and surviving infants. These tests were also used to compare the proportion of infants with each pattern between those with and without morphine during the first 72 h. Kruskal-Wallis test was used to compare the median PED burden between injury severity groups. Binary logistic regression was performed to investigate the relation between the burden of each pattern and injury on cUS and MRI. Linear regression was performed to investigate the relation between the burden of each pattern, the BSITD-III cognitive composite score (uncorrected), the WPPSI-III-NL total IQ, the PED burden, and the Kidokoro global score.

## 3 Results

**Fig. 2:**
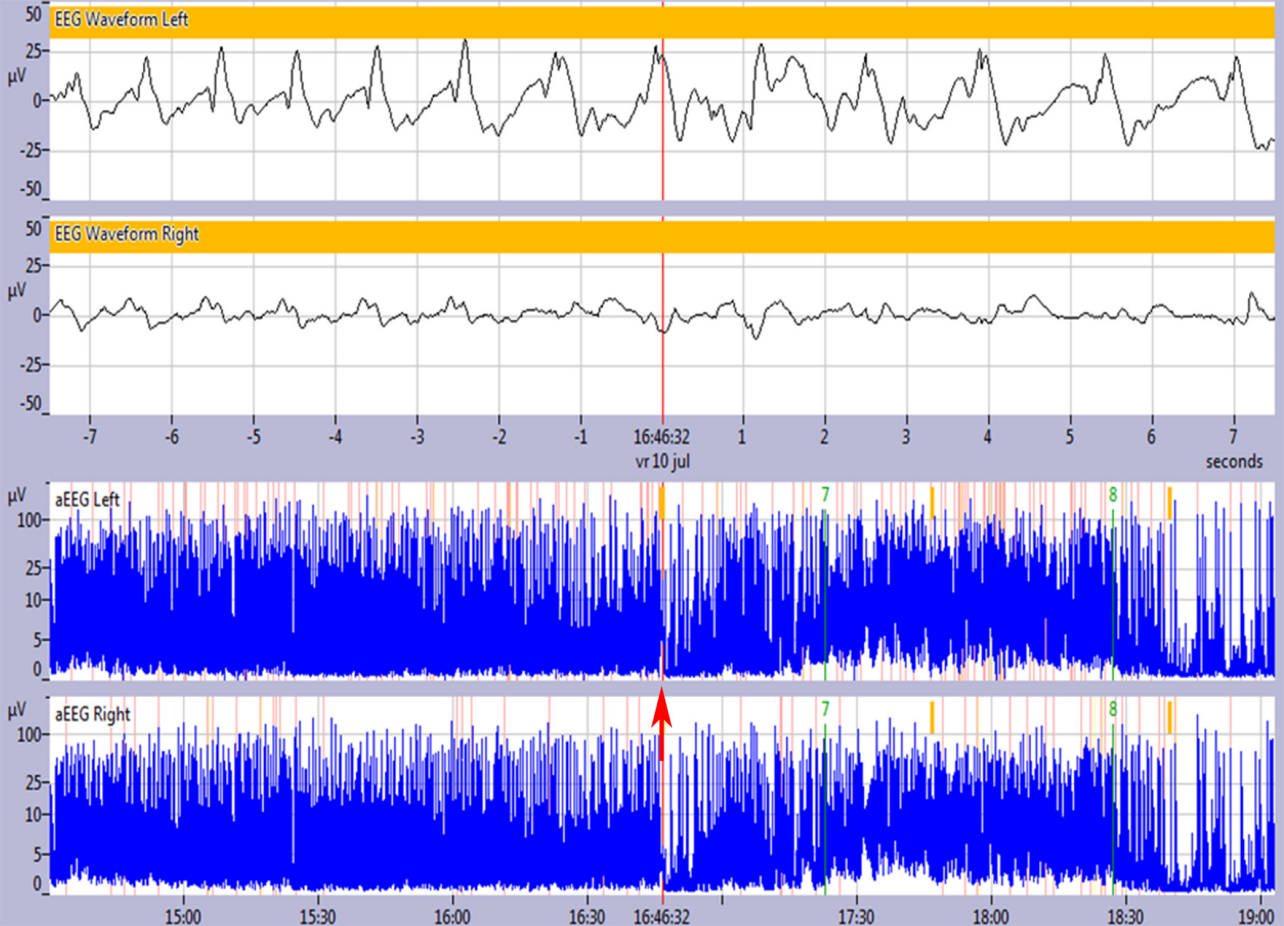
The raw EEG in the upper panel shows an ictal discharge (15 s/screen), with a clear rise of the lower border of the aEEG (arrow) in the lower panel (6 cm/h).

A total of 77 preterm infants were included in this study. During the study period 110 infants were born <28 weeks of gestation. Thirty-three infants were excluded: one was admitted for a very short period, one had a congenital anomaly, 14 had a single channel EEG, 10 had a faulty electrode on one side and in seven infants the EEG file was corrupted. Patient characteristics are shown in Table 1*.* In 29 infants (37.7%), no distinctive rhythmic EEG patterns were observed. The distribution and characteristics of the EEG patterns in the rest of the population are shown in Table 2. Multiple patterns in one infant were observed in 36.4% of the population. Only one infant had ictal discharges during the first 72 h after birth (Fig. 2). This infant was diagnosed with a *Listeria monocytogenes* meningitis. The cUS on admission to the NICU showed fibrin strands in the lateral ventricles and echogenicity in the white matter. Later on, the infant developed intraventricular and parenchymal hemorrhages. The ictal discharges did not respond to antiepileptic therapy and the infant died at day of life 4. Interestingly, the ictal discharges were recognized on aEEG as a clear rise of the lower border, which was never seen in any of the other rhythmic patterns described in this study. Since ictal discharges were only observed in one infant, they were not included in further analyses.

**Table 1:** Patient characteristics.

	Cohort *n* = 77
Sex male/female, *n*	48/29
Gestational age at birth (weeks), median (range)	26.6 (24.5–28.0)
Birth weight (g), median (range)	924 (178)
Birth weight Z-score, mean (SD)	0.37 (0.92)
Small for gestational age (*p* < 10), *n* (%)	5 (6.5)
Apgar at 5 min, median (range)	8 (2–10)
Morphine in first 72 h, *n* (%)	41 (53.2)
Mechanical ventilation, *n* (%)	70 (90.9)

*Neuro-imaging*	
cUS performed, *n* (%)	77 (100)
Abnormal	33 (42.9)
Mild	19 (24.7)
Moderate	5 (6.5)
Severe	9 (11.7)
MRI performed, *n* (%)	68 (88.3)
Abnormal	35 (51.5)
Mild	23 (33.8)
Moderate	9 (13.2)
Severe	3 (4.4)

*Follow-up*	
Died, *n* (%)	7 (9.1)
*Follow-up surviving infants*	
BSITD-III performed, *n* (%)	70 (100)
BSITD-III age (months, uncorrected), mean (SD)	33.1 (0.8)
BSITD-III cognitive composite score (uncorrected), mean (SD)	96 (8)
WPPSI-III-NL performed, *n* (%)	53 (75.7)
WPPSI-III-NL age (years), mean (SD)	5.8 (0.4)
WPPSI-III-NL TIQ, mean (SD)	99 (12)

BSITD-III: Bayley Scales of Infant and Toddler Development, third edition; cUS: cranial ultrasound; MRI: magnetic resonance imaging; SD: standard deviation; TIQ: total intelligence quotient; WPPSI-III-NL: Wechsler Preschool and Primary Scale of Intelligence, third edition, Dutch version.

**Table 2:** Characteristics of the rhythmic EEG patterns.

Pattern	Incidence, *n* (%)	Total duration (s), median (range)	Wavelength (s), mean (SD)
Ictal discharges	1 (1.3)	696 (NA)	0.76 (NA)
PEDs	34 (44.2)	148 (29–1109)	2.07 (0.58)
PED-like waves	22 (28.6)	528 (25–8109)	1.53 (0.68)
Zeta waves	13 (16.9)	212 (57–1729)	1.90 (0.42)
Sinusoidal waves	31 (40.3)	203 (21–32357)	1.12 (0.58)

NA: not applicable; PEDs: periodic epileptiform discharges; SD: standard deviation.

### 3.1 Location EEG pattern versus head position

The EEG location of the rhythmic pattern was significantly associated with head position for the sinusoidal (*p* = 0.038), zeta (*p* = 0.001), and PED-like waves (*p* < 0.001), but not for PEDs (*p* = 0.238). The proportion of events when head position coincided with the presence of sinusoidal, zeta and PED-like waves was 73.7%, for PEDs this was 57.7%.

### 3.2 EEG patterns versus brain injury

**Fig. 3:**
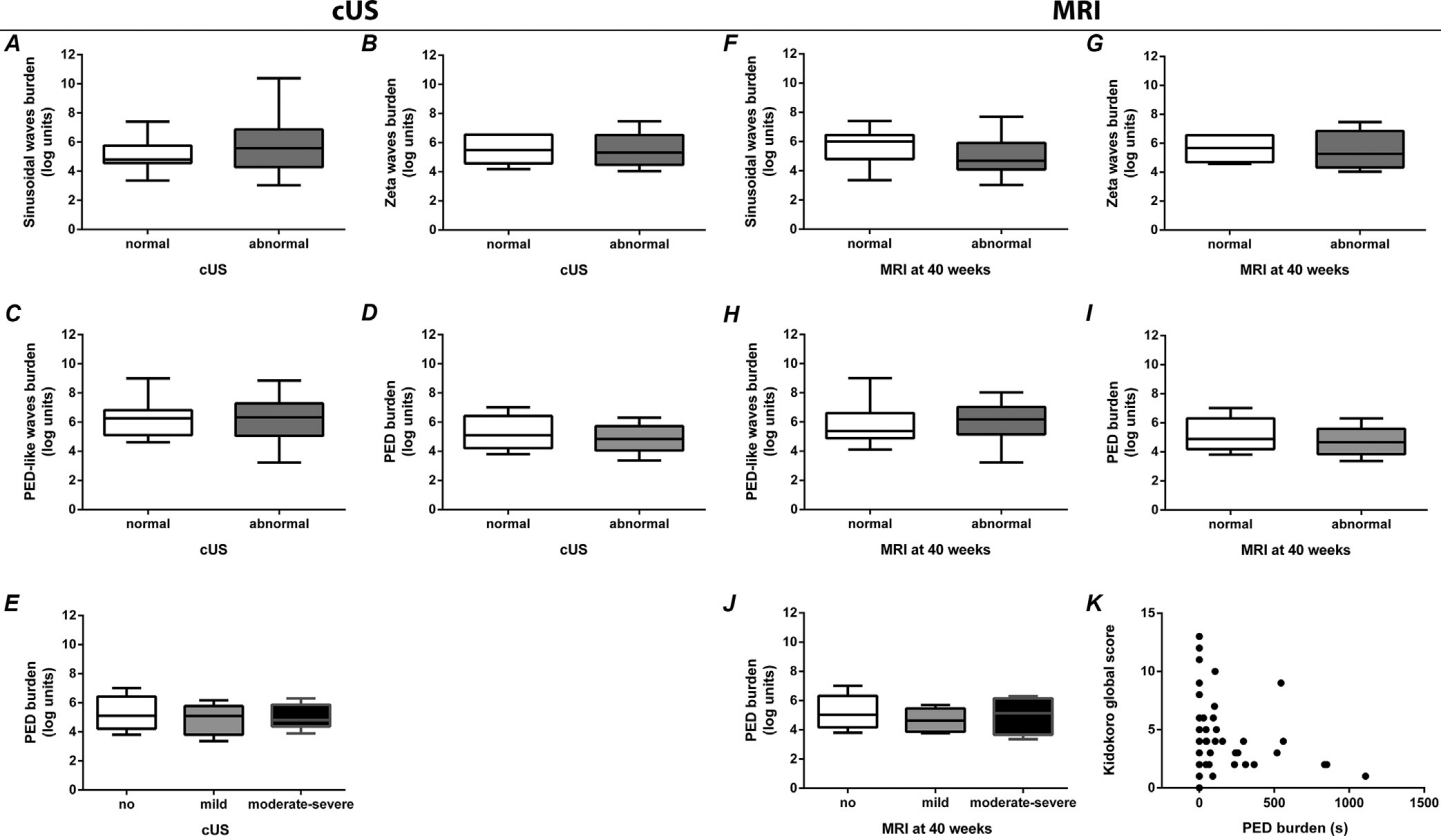
Association between EEG patterns and brain injury on cranial ultrasound (cUS) during the first 72 h (A-E) and MRI at term-equivalent age (F-K). Comparing the log-transformed total duration of each pattern (burden) in those with and without cUS (A–D) or MRI (F–I) abnormalities. (E, J) Comparing the log-transformed PED burden between those with no, mild or moderate-severe injury on cUS (E) and MRI (J). (K) Scatterplot showing the association between the PED burden and the MRI maturation and injury severity score (Kidokoro global score) (Kidokori et al., 2014).

*Sinusoidal, zeta and PED-like waves:* Of the patterns influenced by head position, the proportion of infants with sinusoidal waves was higher in infants with injury on cUS in the first 72 h after birth (*p* = 0.027), but not in infants with injury on MRI at term-equivalent age (*p* = 0.260). Mean suppression intervals during the first 72 h were significantly longer in infants with injury on cUS (12.7 s vs. 9.7 s, *p* = 0.012). The total duration of sinusoidal waves was not related to injury on cUS or MRI. No difference in incidence of zeta or PED-like waves was observed between infants with or without injury on cUS or MRI. The total duration of these patterns was also not related to injury on cUS or MRI (Fig. 3).

*PEDs:* The incidence and total duration of PEDs were not different between infants with and without injury on cUS or MRI. The median PED duration was not different between injury severity groups (no, mild, moderate-severe). No positive relation, but a trend towards a negative relation between total PED duration and MRI injury severity and maturation score was observed (Fig. 3).

### 3.3 EEG patterns versus outcome

*Sinusoidal, zeta and PED-like waves:* Of the patterns influenced by head position, the proportion of infants with sinusoidal (*n* = 6 [85.7%], *p* = 0.015) and PED-like waves (*n* = 5 [71.4%], *p* = 0.018) was significantly higher in infants who died, but no relation was found between the total duration of these patterns and the BSITD-III cognitive composite score at 2 years or IQ at 5 years. No associations were found between the incidence or total duration of zeta waves and any of the outcome parameters.

**Fig. 4:**
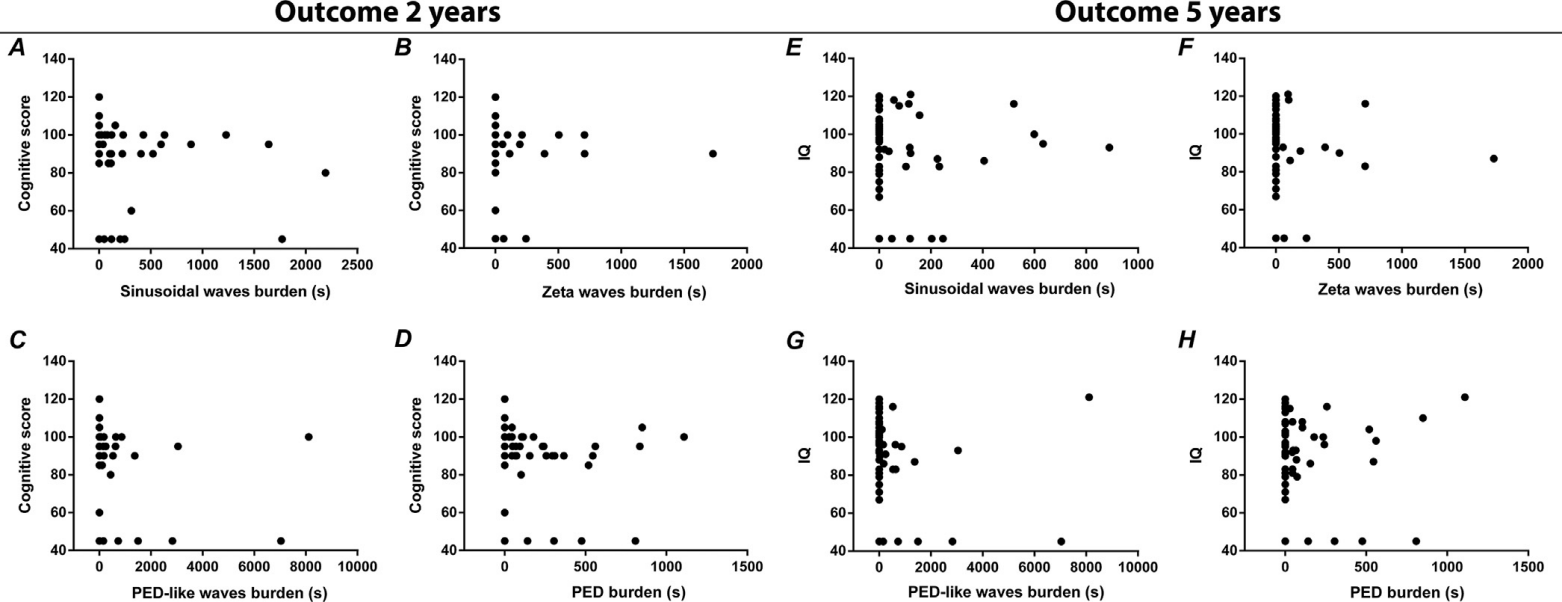
Association between the total duration of the EEG patterns in seconds and the BSITD-III cognitive composite score at 2 years (A–D) and the WPPSI-III-NL total IQ score at 5 years (E–H).

*PEDs:* The incidence and total duration of PEDs was not associated with death, the BSITD-III cognitive composite score at 2 years or IQ at 5 years (Fig. 4)*.*

### 3.4 EEG patterns and the effect of morphine and AEDs

Morphine was administered for sedation during mechanical ventilation at the discretion of the attending neonatologist. The incidence of sinusoidal (+24.3%), zeta (+16.1%), and PED-like waves (+17.1%) was higher while the incidence of PEDs (−11%) was lower in infants who received morphine during the first 72 h compared to infants who did not receive morphine. AEDs were given at the discretion of the attending neonatologist when they suspected an infant to have seizures, either clinical or on EEG. In 11 patients a total of 19 AEDs were administered during the EEG recording (phenobarbital *n* = 11, lidocaine *n* = 4, clonazepam *n* = 2, midazolam *n* = 1, levetiracetam *n* = 1). Seven infants received only one AED, two infants received two different AEDs, one infant three and one received five different AEDs. Four AED administrations were started during a rhythmic pattern, which had a temporary effect on ictal discharges on one occasion, but no effect was seen on sinusoidal, zeta, PED-like waves and PEDs. Fifteen AED administrations were initiated when no rhythmic pattern was observed in the EEG, but in eight of these a rhythmic pattern (sinusoidal, zeta and PED-like waves or PEDs) emerged during administration or shortly thereafter (within 4 h).

## 4 Discussion

In a cohort of 77 extremely preterm infants we found that rhythmic EEG patterns are common during the first 72 h after birth (62.3%). However, clear ictal discharges were only observed in one infant during this period (1.3%). This infant had meningitis and died on day 4 of life. Of the remaining patterns, three (sinusoidal, zeta, and PED-like waves) were significantly related to head position and are likely artefacts. PEDs were not related to head position. They were not related to brain injury or poor cognitive outcome in survivors but their duration had a borderline significant association with brain injury on MRI. This study underlines that the cerebral electrical activity of prematurity has a very specific morphology and that abnormal aspects described in adults and older infants do not have the same prognostic or diagnostic value in extremely preterm infants.

The NICU is an artefact sensitive environment for EEG recording. Appliances like high frequency ventilation or the electrocardiac activity can cause rhythmic artefacts on EEG, especially when the EEG background activity is suppressed. Many artefacts have been described by Andre et al. (2010) However, other machines or actions in the NICU could disturb the EEG signal, causing yet unknown artefacts that are more likely to be picked up with long-term monitoring lasting for several days. The significant relation we found between the location of sinusoidal, zeta, and PED-like waves on EEG and the infant’s head position is striking and strongly suggests that these patterns are artefacts. Sinusoidal and zeta waves have been described in adults and children and were related to serious brain injury and epilepsy, which is contradictive to our results (Magnus and van der Holst, 1987, Watemberg et al., 2007, Watemberg et al., 2002). The frequency of sinusoidal waves was usually around 0.5–1 Hz in our cohort. This could correspond to a respiration and pulse artefact, which usually have a frequency of 60 and 120/min respectively. We found the incidence of sinusoidal waves to be higher in infants with injury on cUS during the first 72 h after birth that subsequently died before an MRI could be made, because this association was no longer seen with MRI at term-equivalent age. These infants had severe brain injury with a very suppressed EEG making them more susceptible to showing artefacts. This suspicion is strengthened by the fact that the incidence of PED-like waves, which also had a strong relation with head position, was also significantly higher in infants who died. PED-like and zeta waves usually had a frequency of 1–2 Hz in our cohort, but we have not been able to relate this to a known artefact. Although, we cannot rule out completely that these patterns are brain activity with pathological significance, since they were not related to head position in all infants, they do not seem to be epileptic in origin, since AEDs did not have an effect on these patterns, and therefore do not warrant immediate treatment with AEDs.

PEDs were the only pattern, apart from ictal discharges, that was not related to head position in our cohort. However, PEDs were also not clearly related to brain injury or poor cognitive outcome in extremely preterm infants. These findings are contradictive to results from adult and pediatric studies, where PEDs have been associated with brain lesions, epilepsy, and poor outcome (Chong and Hirsch, 2005, Hamano et al., 1994, Yemisci et al., 2003). Even in full-term infants they have been associated with herpes simplex encephalopathy, hypoxic-ischemic encephalopathy and stroke in particular (Estivill et al., 1977, Mizrahi and Tharp, 1982, Scher and Beggarly, 1989). It could be postulated that PEDs are a form of spontaneous, hyper-synchronized activity that is related to normal brain development, but should not persist into infancy and childhood. Recent studies have shown that increased brain activity during the first 72 h after birth in the form of bursts or spontaneous activity transients results in faster brain growth in preterm infants (Benders et al., 2014) and that this activity is hyper-synchronized in extremely preterm infants (Benders et al., 2014, Koolen et al., 2016).

The incidence of subclinical electroencephalographic seizures was much lower in our study than previously reported. Scher et al. reported an incidence of 3.9% in infants with a postmenstrual age ≤30 weeks. However, in the study by Scher et al. EEG was only performed when seizures were suspected clinically and this was not restricted to the first 72 h after birth (Scher et al., 1993). Therefore, the a priori chance of seizures was higher. Shah et al., Wikström et al. and Vesoulis et al. recorded infants with a gestational age <30 weeks prospectively during the first 72 h after birth, but reported an incidence of 22–48%, which is much higher than we report (Scher et al., 1993, Vesoulis et al., 2014, Wikström et al., 2012). A possible explanation for this difference could be that PEDs were considered seizures in those reports, since the incidence of PEDs in our cohort (44%) is strikingly similar to their incidence of seizures. PEDs had a similar morphology as ictal discharges in our study, but had a lower frequency and did not show evolution in amplitude, frequency or morphology. Also, no change in the aEEG trace was observed as opposed to ictal discharges. Ictal discharges in preterm infants have been reported to be lower in frequency and shorter in duration. Different patterns of evolution in frequency such as acceleration and unchanged frequency as opposed to deceleration have been reported as well in preterm infants (Janachova et al., 2016). In this respect PEDs could be considered as ictal discharges. However, PEDs were not related to brain injury in our cohort and outcome was normal independent of high PED burden. Patterns resembling PEDs that do not show evolution on 2-channel EEG should therefore be considered with caution. They should not immediately be marked as seizures and treatment should be withheld until subclinical electroencephalographic seizures are confirmed on multichannel EEG. Especially, since AEDs did not have an effect on PEDs in our cohort.

It should be noted that this study has some limitations. First, we only assessed the EEG at the markers placed by the seizure detection algorithm of the BrainZ monitor (Navakatikyan et al., 2006). We chose to do so because in everyday clinic, assessment of long-term EEG recordings is mostly limited to the parts where markers are placed by the seizure detection algorithm and parts with changes in the aEEG trace. However, the BrainZ algorithm has a sensitivity of 83–95% for detecting seizures in full-term infants, and it is likely that the algorithm did not detect all rhythmic EEG patterns and the true burden of these patterns may be higher than reported in this study (Navakatikyan et al., 2006). The sensitivity in extremely preterm infants and the sensitivity for non-seizure rhythmic patterns are unknown. Second, the EEG assessments were made with continuous 2-channel EEG without extra polygraphic variables such as electrocardiogram and respiration. Multichannel video-EEG remains the gold standard for detection and quantification of rhythmic patterns and seizures (Shellhaas et al., 2011). It can localize the different patterns to certain areas and when polygraphy is used common artefacts such as respiration can easily be ruled out. Furthermore, experienced technicians will detect movement artefacts and artefacts due to inadequate electrode impedance.

Our results show that automatic seizure detection algorithms should be used with caution, especially in preterm infants. The seizure detection algorithm on the BrainZ monitor has not been validated for these extremely preterm infants. If detections are not reviewed carefully it may result in unnecessary AED administration and an infant might be incorrectly identified as having seizures. This will cause unnecessary concern for the parents and AEDs may be harmful to the developing brain as well. It should also be noted that electrodes other than needle electrodes may give rise to even more artefacts. Clinicians faced with rhythmic EEG patterns in 2-channel EEG of extremely preterm infants should first place the infant’s head in the midline and make sure the electrodes are free from contact with surrounding materials and be placed correctly. This will eliminate several artefacts. If rhythmicity is still observed, only when clear evolution in amplitude, frequency or morphology is visible (best visible when 10 s/screen is changed to 1 min/screen), treatment with AEDs should be considered. The aEEG can aid in distinguishing PEDs from ictal discharges, showing a simultaneous rise in the lower border of the aEEG with ictal discharges. Patterns resembling sinusoidal, zeta or PED-like waves do not need treatment. When PEDs are observed, a multichannel video-EEG should be performed to confirm or rule out ictal discharges. Further research using multichannel video-EEG is needed to investigate the significance of PEDs in preterm brain development.

## 5 Conclusion

Rhythmic EEG patterns are common in extremely preterm infants, but ictal discharges were only observed in 1.3%. Furthermore, three patterns (sinusoidal, zeta and PED-like waves) were significantly related to head position and are likely artefacts. PEDs were common and not related to head position, but were also not associated with brain injury or poor cognitive outcome. This study shows that EEG patterns described in older infants and adults do not have the same prognostic or diagnostic value in extremely preterm infants. Clinicians using the BrainZ seizure detection algorithm in extremely preterm infants should review rhythmic activity marked by the algorithm carefully before starting AED treatment.

## Ethical publication statement

We confirm that we have read the Journal’s position on issues involved in ethical publication and affirm that this report is consistent with those guidelines.

## Disclosure statement

None of the authors has any conflict of interest to disclose.
